# Using machine learning to predict venous thromboembolism and major bleeding events following total joint arthroplasty

**DOI:** 10.1038/s41598-022-26032-1

**Published:** 2023-02-07

**Authors:** Noam Shohat, Leanne Ludwick, Matthew B. Sherman, Yale Fillingham, Javad Parvizi

**Affiliations:** 1 Rothman Orthopaedic Institute at Thomas Jefferson University, 125 S 9th St. Ste 1000, Philadelphia, PA 19107 USA; 2 Sackler Faculty of Medicine, Tel Aviv University, Ramat Aviv, Israel

**Keywords:** Orthopaedics, Outcomes research

## Abstract

Venous thromboembolism (VTE) and major bleeding (MBE) are feared complications that are influenced by numerous host and surgical related factors. Using machine learning on contemporary data, our aim was to develop and validate a practical, easy-to-use algorithm to predict risk for VTE and MBE following total joint arthroplasty (TJA). This was a single institutional study of 35,963 primary and revision total hip (THA) and knee arthroplasty (TKA) patients operated between 2009 and 2020. Fifty-six variables related to demographics, comorbidities, operative factors as well as chemoprophylaxis were included in the analysis. The cohort was divided to training (70%) and test (30%) sets. Four machine learning models were developed for each of the outcomes assessed (VTE and MBE). Models were created for all VTE grouped together as well as for pulmonary emboli (PE) and deep vein thrombosis (DVT) individually to examine the need for distinct algorithms. For each outcome, the model that best performed using repeated cross validation was chosen for algorithm development, and predicted versus observed incidences were evaluated. Of the 35,963 patients included, 308 (0.86%) developed VTE (170 PE’s, 176 DVT’s) and 293 (0.81%) developed MBE. Separate models were created for PE and DVT as they were found to outperform the prediction of VTE. Gradient boosting trees had the highest performance for both PE (AUC-ROC 0.774 [SD 0.055]) and DVT (AUC-ROC 0.759 [SD 0.039]). For MBE, least absolute shrinkage and selection operator (Lasso) analysis had the highest AUC (AUC-ROC 0.803 [SD 0.035]). An algorithm that provides the probability for PE, DVT and MBE for each specific patient was created. All 3 algorithms had good discriminatory capability and cross-validation showed similar probabilities comparing predicted and observed failures indicating high accuracy of the model. We successfully developed and validated an easy-to-use algorithm that accurately predicts VTE and MBE following TJA. This tool can be used in every-day clinical decision making and patient counseling.

## Introduction

More than 540,000 total knee arthroplasties (TKA) and 230,000 total hip arthroplasties (THA) are annually performed in the United States, and these numbers continue to rise steadily^1,2^. Rates of venous thromboembolism (VTE) that consist of deep venous thrombosis (DVT) and pulmonary embolism (PE) range between 1 and 3% depending on a host of factors^3,4^. VTE is associated with immense morbidity and increased cost for episode of care and therefore has attracted much attention in recent years and with the introduction of bundled care^5,6^.

There are numerous risk factors for development of VTE that are either host or surgery related^7–12^. While it is important to know the individual risk factors associated with VTE, that alone does not always contribute to clinical decision making and overall risk stratification. In the era of personalized medicine, and taking into consideration the many existing pharmacological and non-pharmacological options for VTE prophylaxis, individualized risk scores are desperately needed. The Caprini score that is utilized in other surgical fields has never been validated on orthopedic surgical patients and is not applicable to patients undergoing TJA^13^. Subsequently—numerous risk stratification models have been suggested to be used following total joint arthroplasty (TJA), and while moving towards a more personalized approach for risk stratification they lack proper validation^8,14–16^.

Besides VTE, the risk of major bleeding events (MBE) in the postoperative period can also be consequential and occasionally fatal^17–22^. While similar rates of VTE and MBE are expected, the latter has received much less focus^23,24^. As evidence, none of the previous VTE scores take MBE into consideration, and as far as we are aware, there is currently no risk stratification model for MBE. VTE risk stratification and the influence on prevention modalities such as chemoprophylaxis have a direct influence on MBE^17–22,25–28^. Understanding this relationship may aid in providing an ideal risk‐benefit ratio to decide on the optimal VTE prophylaxis.

Many developments have occurred in the last decade in the field of joint arthroplasty including faster recovery and early mobilization, the use of tranexamic acid, spinal anesthesia, and the transition to aspirin for prophylaxis^29–31^. These factors have positively impacted the outcome of joint arthroplasty by minimizing complications and facilitating rapid recovery, to the point that some are now done as outpatient procedures^32^. In parallel with these changes in surgery, in recent years machine learning has been introduced into many areas within the healthcare system with the potential to revolutionize the medical landscape^33–35^. Recent developments in machine learning have facilitated a more comprehensive, accurate and user-friendly platform that may help clinicians in decision making.

Using a contemporary large institutional database with granular data, this study aimed to develop and validate an algorithm suitable for use in everyday clinical practice that could predict the probability of developing VTE and MBE in TJA patients, taking into account the influence of a large number of variables.

## Methods

This was a single institution, retrospective cohort study. All methods were carried out in accordance with relevant guidelines and regulations. This study was reviewed and approved by the Institutional Review Board of Thomas Jefferson University with a waiver of informed consent. Following IRB approval, medical records of 37,948 patients who underwent either primary or revision total hip or knee arthroplasty (THA or TKA) between January 2009 and October 2020 were reviewed. STROBE reporting guidelines were followed throughout the data collection process^36^. Patients for whom a minimum 90-day follow-up was not available were excluded leaving us with 35,963 cases included in the study.

Sex, age, race, body mass index (BMI), patient-reported past medical history, Charlson Comorbidity and Elixhauser Comorbidity indexes were broken down to their individual components, as well as the American Society of Anesthesiologists (ASA) classification were collected. Variables that have shown an association with VTE in previous publications including hormone replacement therapy, rheumatoid arthritis, Sjogren’s syndrome, lupus, varicose veins, irritable bowel syndrome, history of stroke, myeloproliferative disease, and sleep apnea, were queried utilizing International Classification of Disease (ICD)-9 and ICD-10 codes^37^. Patients with any type of active cancer or a history of cancer were also identified and stratified based on a previously published VTE predictive model developed by Khorana et al.^38,39^. Keyword searches were conducted to identify coagulopathies and hypercoagulability in the patient population (Supplementary Table 1–2). Notes containing a keyword for coagulopathy or hypercoagulability (n = 74,886) were isolated and reviewed to enhance capture rates.

Clinical notes, hospital orders, and discharge summaries were reviewed to determine VTE prophylaxis prescribed to each patient postoperatively as well as identify chronic anticoagulation that the patient may be taking preoperatively. These were grouped into distinct groups—none, aspirin 81 mg (twice daily), aspirin 325 mg (twice daily), warfarin and others (including anti-factor Xa, Unfractionated Heparin, low molecular weight heparin, fondaparinux, Adenosine diphosphate receptor inhibitor and direct thrombin inhibitor). Information regarding the operation, including the specific joint operated on (knee versus hip), whether it was unilateral or bilateral, operative time, tourniquet use for only TKA procedures, surgical approach for only THA procedures, surgeon volume (dichotomized to normal versus high), use of cement, tranexamic acid administration, intraoperative blood transfusions (divided into 3 distinct categories: no transfusion, 1 unit transfusion and 2 or more unit transfusion) as well as type of anesthesia (regional versus general) was also collected from operative reports and anesthesia logs.

Two distinct outcomes were evaluated. The first was the occurrence of symptomatic DVT or PE within 90 days of surgery. To avoid including superficial clots that were not clinically significant, only patients that had a documented diagnosis, confirmatory study, and treatment for VTE were considered to have met the primary endpoint. The second main outcome was occurrence of major bleeding events (MBE) as defined by the Scientific and Standardization Committee of the International Society on Thrombosis and Haemostasis^40^. Symptomatic VTE and MBE occurring within 90 days of the operation were identified from medical records. To enhance the capture rate, comprehensive queries utilizing keywords for DVT, PE and MBE were conducted in clinical notes, physician dictations, and patient-provider phone-call logs (Supplementary Table 3 and 4). Notes containing a keyword for DVT (n = 44,752), PE (n = 14,878) and MBE (n = 9149) were isolated and manually reviewed. All readmissions within 90 days were also reviewed to detect any uncaptured VTE or MBE event.

### Statistical analysis

Prior to running the predictive algorithms, a set of descriptive statistics were performed to understand the data distributions. Patients with VTE were compared to those who did not have VTE and those with MBE were compared to those who did not have MBE. Continuous data is presented as a mean (standard deviation) and categorical data is presented as a cell count (%). T-tests were used to calculate *p* values for continuous data and Chi-Square tests were used to calculate *p* values for categorical data. Due to the nature of comparisons in the first table, the alpha was adjusted to 0.001.

Following the descriptive breakdown, various machine learning methods were applied with the main objective of being able to determine specific variables that produced an increase likelihood chance of getting a DVT, PE, or MBE. The four models that were tested where Random Forest (RF), LASSO, Gradient Boosting Trees (XGB), and Support Vector Machines (SVM). Due to the nature of imbalance in the data as well, some of the models were also performed using down-sampling. In order to validate each model, both the VTE and MBE data sets were split out into a 70–30% split so we could properly train the data and test it.

Each training model analyzed used repeated cross validation (CV) techniques, where one fold was removed each time the model was fitted. The repeated CV was done 3 times with fivefold each time. To determine the best models from training, AUCs and Precision Curves were calculated. Once the “best” model was selected, the remaining data (test data) was tested on it to ensure it had the proper performance. All statistical analyses were done using R Studio (Version 3.6.3, Vienna, Austria).

## Results

Of the 35,963 patients included in the study, 308 (0.86%) developed VTE (170 PE’s, 176 DVT’s) and 293 (0.81%) developed MBE. There were significant differences in patient demographics, characteristics, comorbidities, anticoagulation medications and operative factors between patients who developed VTE and MBE and those who did not (Table 1).

**Table 1:** Patient demographics, characteristics, operative and anticoagulation medications stratified based on development of venous thromboembolism and major bleeding events.

	VTE			MBE		
	No VTE (n = 35,655)	VTE (n = 308)	*p*-value	No MBE (n = 35,670)	MBE (n = 293)	*p*-value
**Preoperative Chronic Anticoagulation**						
None	17,536 (67.7%)	156 (0.9%)^$^	< 0.001	17,546 (67.8%)	146 (0.8%)^$^	< 0.001
Aspirin	5959 (23.0%)	62 (1.0%)^$^		5942 (23.0%)	79 (1.3%)^$^	
Warfarin	937 (3.6%)	12 (1.3%)^$^		933 (3.6%)	16 (5.7%)^$^	
Other	1468 (5.7%)	32 (2.1%)^$^		1458 (5.6%)	42 (2.8%)^$^	
**Demographics and Habits**						
Age, years	63.8 (10.9)	67.1 (9.4)	< 0.001	63.8 (10.94)	67.7 (11.57)^$^	< 0.01
Gender (male)	16,484 (45.8%)	149 (48.1%)*	0.457	16,486 (45.8%)	147 (51.0%)*	0.085
Race						
White	23,897 (78.3%)	173 (0.7%)^$^	0.115	23,882 (78.3%)	188 (0.8%)^$^	0.802
African American	3925 (12.9%)	41 (1.0%)^$^		3935 (12.9%)	31 (0.8%)^$^	
Other	2687 (8.8%)	23 (0.8%)^$^		2692 (8.8%)	18 (0.7%)^$^	
BMI (kg/m^2^)	30.3 (38.5)	31.4 (6.0)	0.008	30.3 (38.5)	31.89 (8.10)	0.002
Smoking						
No	20,205 (59.4%)	171 (0.8%)^$^	0.057	20,237 (59.5%)	139 (0.7%)^$^	< 0.001
Current	2978 (8.8%)	17 (0.6%)^$^		2958 (8.7%)	37 (1.2%)^$^	
Past	10,847 (31.9%)	109 (1.0%)^$^		10,840 (31.8%)	116 (1.1%)^$^	
Alcohol						
No	15,081 (44.1%)	154 (1.0%)^$^	0.033	15,086 (44.1%)	149 (1.0%)^$^	0.030
Occasional	16,587 (48.5%	126 (0.8%)^$^		16,584 (48.5%)	129 (0.8%)^$^	
Heavy	2499 (7.3%)	18 (0.7%)^$^		2503 (7.3%)	14 (0.6%)^$^	
**Previous History and Comorbidities**						
History of VTE	1468 (4.1%)	27 (8.6%)*	< 0.001	1457 (4.0%)	38 (12.9%)*	< 0.001
Hormone Replacement Therapy	1396 (3.9%)	303 (3.5%)*	0.883	1401 (3.9%)	6 (2.0%)*	0.126
Hyper-coagulopathy	536 (1.5%)	18 (5.7%)*	< 0.001	538 (1.5%)	16 (5.4%)*	< 0.001
ASA Score						
0	588 (1.7%)	5 (0.8%)^$^	< 0.001	590 (1.7%)	3 (0.5%)^$^	< 0.001
1	882 (2.5%)	1 (0.1%)^$^		883 (2.5%	0 (0.0%)^$^	
2	18,330 (52.9%)	108 (0.6%)^$^		18,352 (52.9%)	86 (0.5%)^$^	
3	14,576 (42.1%)	177 (1.2%)^$^		14,557 (42.0%)	196 (1.3%)^$^	
4	279 (0.8%)	6 (2.1%)^$^		278 (0.8%)	7 (2.5%)^$^	
AIDS	24 (0.1%)	0 (0.0%)*	1.000	24 (0.1%)	0 (0%)*	1.000
Myocardial Infarct	1298 (3.8%)	17 (5.6%)*	0.129	1289 (3.7%)	26 (8.9%)*	< 0.001
Heart Failure	760 (2.2%)	16 (5.2%)*	0.002	755 (2.2%)	21 (7.2%)*	< 0.001
Other cardiovascular disease	465 (1.3%)	11 (3.6%)*	0.004	468 (1.4%)	8 (2.7%)*	0.066
Atrial Fibrillation	3352 (9.3%)	50 (16.0%)*	< 0.001	3344 (9.3%)	58 (19.7%)*	< 0.001
Chronic Obstructive Pulmonary Disease	4004 (11.6%)	52 (17.0%)*	0.004	4022 (11.6%)	34 (11.6%)*	0.991
Dementia	77 (0.2%)	3 (1.0%)*	0.034	78 (0.2%)	2 (0.7%)*	0.145
Diabetes Mellitus						
No	32,793 (95.0%)	295 (0.9%)^$^	0.021	32,809 (95.0%)	279 (0.8%)^$^	0.001
Simple	1380 (4.0%)	5 (0.4%)^$^		1381 (4.0%)	4 (1.4%)^$^	
Complicated (organ damage)	343 (1.0%)	6 (2.0%)^$^		340 (1.0%)	9 (3.1%)^$^	
Hemiparesis	31 (0.1%)	0 (0.0%)*	1.000	30 (0.1%)	1 (0.3%)*	0.230
Liver Disease	342 (1.0%)	5 (1.4%)*	0.238	344 (1.0%)	3 (1.0%)*	0.768
Peptic Ulcer Disease	128 (0.4%)	3 (1.0%)*	0.109	122 (0.4%)	9 (3.1%)*	< 0.001
Peripheral Vascular Disease	537 (1.6%)	7 (2.3%)*	0.346	537 (1.6%)	7 (2.4%)*	0.231
Chronic renal failure	957 (2.8%)	21 (6.9%)*	< 0.001	958 (2.8%)	20 (6.8%)*	< 0.001
Inflammatory Arthritis						
None	33,271 (96.4%)	297 (0.9%)^$^	0.263	33,293 (96.4%)	275 (0.8%)^$^	0.015
Rheumatoid arthritis (RA)	1036 (3.0%)	6 (0.6%)^$^		1031 (3.05)	11 (1.1%)^$^	
Sjogren	63 (0.2%)	0 (0%)^$^		59 (0.2%)	4 (6.3%)^$^	
Lupus Erythematous	146 (0.4%)	3 (1.0%)^$^		147 (0.4%)	2 (1.3%)^$^	
Chronic Anemia	2058 (5.7%)	30 (9.6%)*	0.005	2060 (5.7%)	28 (9.5%)*	0.009
Varicose Veins	107 (0.3%)	1 (0.3%)*	0.609	102 (0.3%)	6 (2.0%)*	< 0.001
Irritable Bowel Syndrome	120 (0.3%)	2 (0.6%)*	0.284	122 (0.3%)	0 (0.0%)*	1.000
Cerebrovascular Accident	247 (0.7%)	1 (0.3%)*	0.729	248 (0.7%)	0 (0.0%)*	0.275
Sleep Apnea	1029 (2.9%)	11 (3.5%)*	0.493	1037 (2.9%)	3 (1.0%)*	0.053
**Malignancy**						
Active non-metastatic Malignancy						
None	34,651 (96.2%)	268 (0.8%)^$^	< 0.001	34,663 (96.2%)	256 (0.7%)^$^	< 0.001
Baseline risk	311 (0.9%)	5 (1.6%)^$^		308 (0.9%)	8 (2.5%)^$^	
High Risk	1013 (2.8%)	35 (3.3%)^$^		1021 (2.8%)	27 (2.6%)^$^	
Very High Risk	43 (0.1%)	6 (12.2%)^$^		46 (0.1%)	3 (6.1%)^$^	
History of Cancer						
None	32,705 (90.8%)	288 (0.9%)^$^	0.760	32,745 (90.9%)	248 (0.8%)^$^	< 0.001
Baseline risk	41 (7.6%)	23 (0.8%)		2725 (7.6%)	39 (1.4%)^$^	
High Risk	554 (1.5%)	3 (1.0%)^$^		551 (1.5%)	6 (1.1%)^$^	
Very High Risk	18 (0.0%)	0 (0.0%)^$^		17 (0.0%)	1 (5.6%)^$^	
Metastatic Disease	97 (0.3%)	5 (1.6%)*	0.002	100 (0.3%)	2 (0.7%)*	0.211
Myeloproliferative Disease	32 (0.1%)	1 (0.3%)*	0.249	32 (0.1%)	1 (0.3%)*	0.235
**Operative factors**						
Joint (knee)	15,711 (47.3%)	97 (66.4%)*	< 0.001	15,769 (47.4%)	134 (48.4%)*	0.763
Simultaneous Bilateral	1565 (5.1%)	28 (10.3%)*	< 0.001	1582 (5.2%)	11 (4.3%)*	0.669
Underlying Fracture	337 (0.9%)	14 (4.5%)*	< 0.001	343 (1.0%)	8 (2.7%)*	0.008
Operative Duration (minutes)	79.6 (38.1)	92.9 (52.2)*	< 0.001	79.5 (38.0)	106.0 (60.7)*	< 0.001
Revision Surgery	5358 (14.9%)	81 (25.8%)*	< 0.001	5285 (14.7%)	154 (52.4%)*	< 0.001
Cemented Prosthesis	14,111 (39.2%)	174 (55.4%)*	< 0.001	14,130 (39.2%)	155 (52.7%)*	< 0.001
Tourniquet use (knees only)	11,227 (71.2%)	146 (76.0%)*	0.151	11,256 (71.2%)	117 (87.3%)*	< 0.001
Surgical approach (Hips only)						
Direct Anterior	7227 (41.1%)	25 (0.3%)^$^	0.002	7231 (41.2%)	21 (0.3%)^$^	< 0.001
Direct Lateral	10,350 (58.9%)	73 (0.7%)^$^		10,301 (58.8%)	122 (1.2%)^$^	
Surgeon Volume (high)	30,433 (87.7%)	49 (83.6%)*	0.039	30,475 (87.8%)	208 (71.2%)*	< 0.001
Spinal Anesthesia	31,871 (92.4%)	249 (83.8%)*	< 0.001	31,915 (92.5%)	205 (70.4%)*	< 0.001
Tranexamic use	16,160 (46.5%)	85 (29.8%)*	< 0.001	16,167 (46.5%)	78 (30.0%)*	< 0.001
Blood Transfusion						
No	34,003 (94.4%)	248 (0.7%)^$^	< 0.001	34,032 (94.4%)	219 (0.6%)^$^	< 0.001
Single unit	1106 (3.1%)	30 (2.6%)^$^		1110 (3.1%)	26 (2.3%)^$^	
2 or more units	909 (2.5%)	36 (11.5%)^$^		896 (2.5^)	49 (5.2%)^$^	
**Postop Anticoagulation Medication**						
Warfarin	11,189 (31.4%)	161 (1.4%)^$^	< 0.001	11,222 (31.5%)	128 (1.1%)^$^	< 0.001
Other	2954 (8.3%)	34 (1.1%) ^$^		2958 (8.3%)	30 (1.0%)^$^	
Aspirin	21,512 (60.3%)	113 (0.5%)^$^		21,490 (60.2%)	135 (0.6%)^$^	
Aspirin 81 mg	11,191 (57.7%)	52 (0.5%)^$^	0.191	11,192 (57.7%)	51 (0.5%)^$^	0.001
Aspirin 325 mg	8211 (42.3%)	50 (0.6%)^$^		8193 (42.3%)	68 (0.8%)^$^	

Categorical variables were analyzed with a Chi square test and continuous variables with a Student's t-test. An alpha of 0.05 was used to evaluate significance. Data presented as number (percentage) or mean (standard deviation). *Percent within VTE/MBE. ^$^Percent within the tested variable. American Society of Anesthesiologists (ASA); Milligram (mg); Kilogram (kg); Meter (m); Venous thromboembolism (VTE); Major bleeding event (MBE).

### Venous thromboembolism

Risk factors for VTE in the initial univariate analysis were preoperative use of chronic anticoagulation other than aspirin (*p* < 0.001), older age (*p* < 0.001), higher BMI (*p* = 0.008), history of VTE (*p* < 0.001), hyper-coagulopathy (*p* < 0.001), higher ASA score (*p* < 0.001), heart failure (*p* = 0.002), atrial fibrillation (*p* < 0.001), other cardiovascular disease (*p* = 0.004), COPD (*p* = 0.004), dementia (*p* = 0.034), complicated DM (*p* = 0.021), CRF (*p* < 0.001), chronic anemia (*p* = 0.005), active malignancy (*p* < 0.001), metastatic disease (*p* = 0.002), knee joint (*p* < 0.001), simultaneous bilateral surgery (*p* < 0.001), underlying fracture (*p* < 0.001), operative duration (0.001), revision surgery (*p* < 0.001), cemented prosthesis (*p* < 0.001), direct lateral approach to the hip (*p* = 0.002), general anesthesia (*p* < 0.001), not using tranexamic acid (*p* < 0.001), allogenic blood transfusions (*p* < 0.001) and use of Warfarin for VTE prophylaxis (*p* < 0.001).

**Figure 1:**
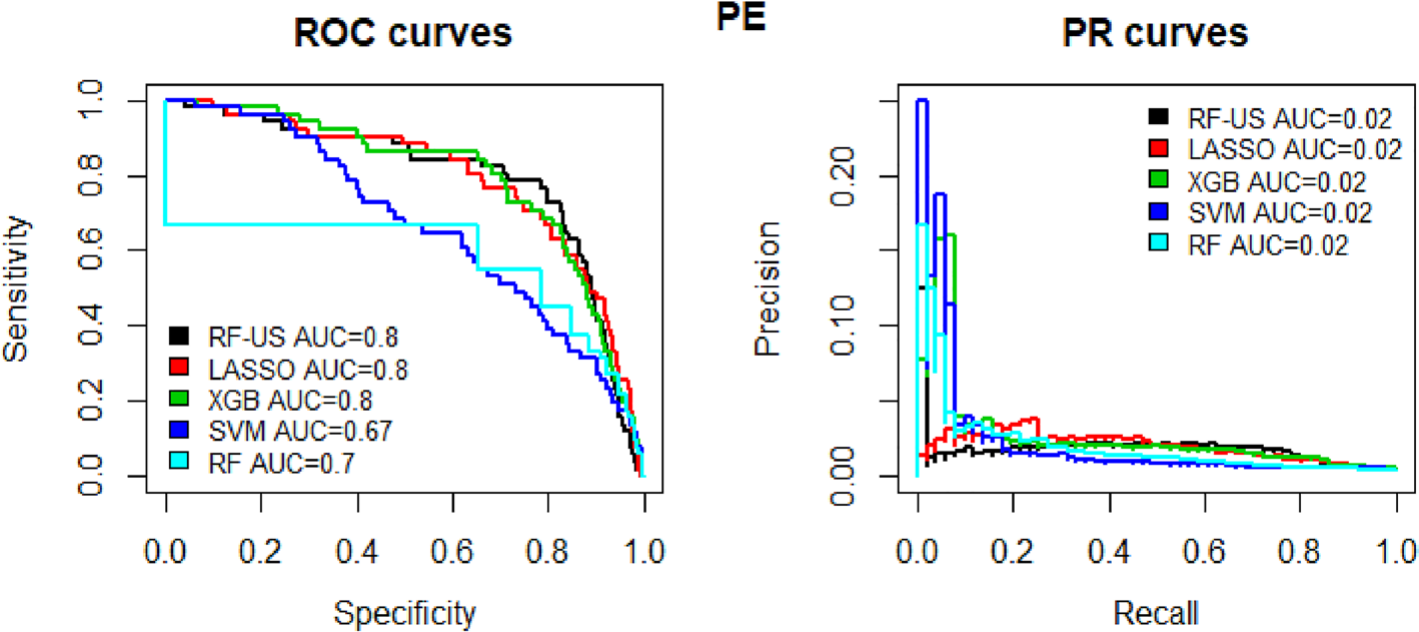
ROC curves for the various models that were assessed for PE.

**Figure 2:**
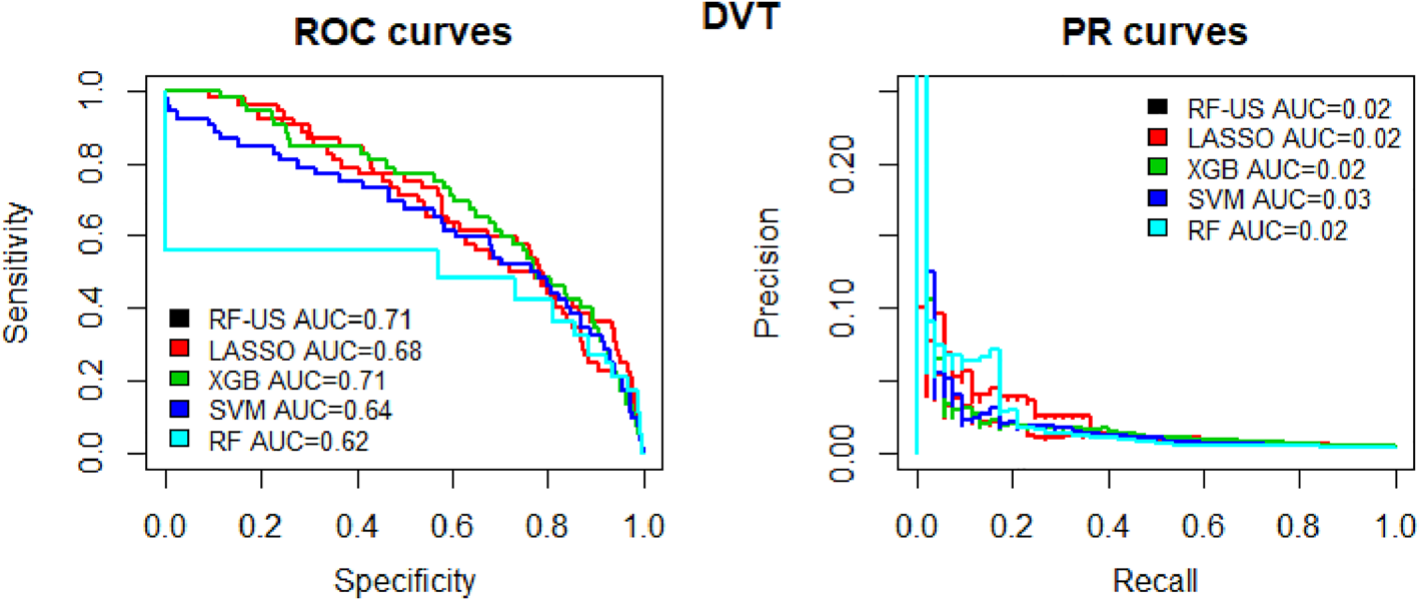
ROC curves for the various models that were assessed for DVT.

**Figure 3:**
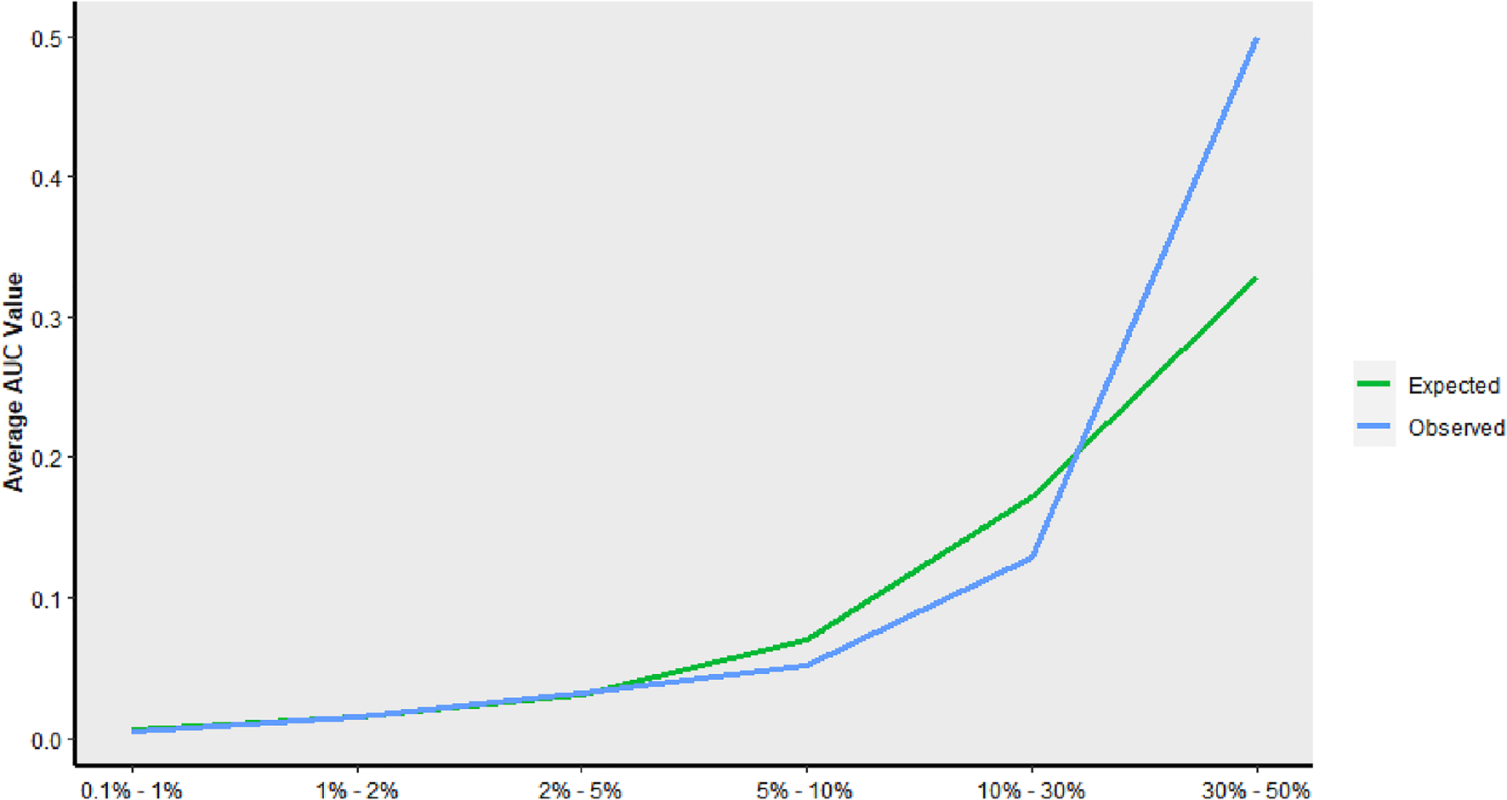
Predicted probability of venous thromboembolism (pulmonary embolus or deep vein thrombosis) and observed proportion stratified into groups based on risk.

Separate models were developed for DVT and PE prediction and were tested using repeated cross-validation. Gradient boosting trees had the highest performance for both PE (AUC-ROC 0.774 [SD 0.055]) and DVT (AUC-ROC 0.759 [SD 0.039]) prediction and was chosen for algorithm development (Figs. 1 and 2 reflect performance on the validation cohort). Patients were grouped into categories according to predicted probability of VTE and the proportion of actual VTE in each category was examined; high agreement between expected and observed events was seen (Fig. 3).

**Figure 4:**
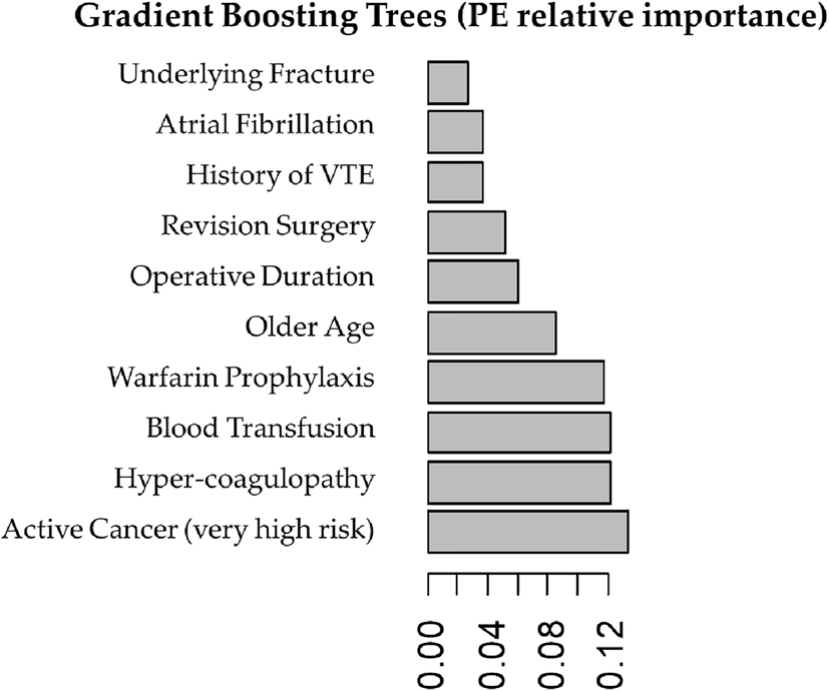
Top ten important predictors for pulmonary embolus (PE) as captured by gradient boosting tree analysis.

**Figure 5:**
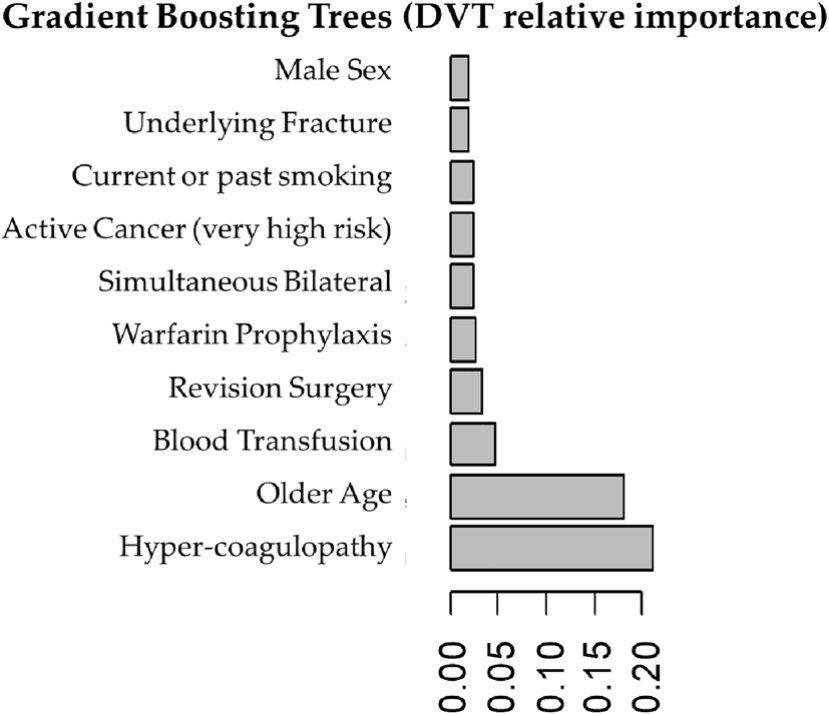
Top ten important predictors for deep vein thrombosis (DVT) as captured by gradient boosting tree analysis.

Gradient boosting trees analysis showed the 10 most important factors associated with PE were the following (by order of importance): active cancer (very high risk), hyper-coagulopathy, blood transfusion, Warfarin for VTE prophylaxis, older age, operative duration, revision surgery, history of VTE, atrial fibrillation and underlying fracture (Fig. 4). For DVT, the 10 most important factors were the following (by order of importance): hyper-coagulopathy, older age, allogenic blood transfusions, revision surgery, Warfarin prophylaxis, simultaneous bilateral surgery, active cancer (very high risk), active or former smoking, underlying fracture and male sex (Fig. 5). Examples of different clinical scenarios and the algorithm predictions are demonstrated in Table 2.

**Table 2:** Example patient characteristics, expected probability for failure and actual outcome.

Age, sex, BMI	Risk factors	Operative/perioperative details (surgery, operation time, anesthesia, TXA, allogenic blood transfusion)	VTE Prophylaxis	Probability for VTE	Probability for MBE (%)	Outcome
66 yr, M, 34.7 kg/m^2^	Former smoker, liver disease, PUD, varicose veins, past cancer	Primary THA, 81 min, general anesthesia, no TXA, 2 RBC units	Warfarin	6.0% (PE) 3.8% (DVT)	57.6	MBE
55 yr, F, 17.4 kg/m^2^	Previous VTE, CHF, PUD, CRF, active cancer (high risk)	Revision THA, 113 min, general anesthesia, no TXA, 2 RBC units	Warfarin	2.2% (PE) 2.1% (DVT)	43.5	MBE
85 yr, M, 24.6 kg/m^2^	Former smoker, Chronic anticoagulation (warfarin), Hypercoagulobality, CHF, Atrial fibrillation, PUD, CRF, CVA, active cancer (baseline risk),	primary TKA, 128 min, spinal anesthesia, no TXA, 2 RBC units	Warfarin	11.7% (PE) 54.7% (DVT)	12.4	DVT
80 yr, M, 30.3 kg/m^2^	Atrial Fibrillation, CRF. Active cancer (high risk),	Revision TKA, 168, spinal anesthesia, no TXA, 2 RBC units	Warfarin	11.8% (PE) 22.2% (DVT)	6.8	DVT and PE
49 yr, F, 25.5 kg/m^2^	Current smoker, , Chronic anticoagulation, history of VTE	THA for fracture, 91 min, general anesthesia, no TXA	Dabigatran	3.6% (PE) 2.8 (DVT)	3.4	PE
63 yr, F, 34.5 kg/m^2^	Chronic aspirin, CHF, CRF, active cancer (highest risk)	Primary TKA, 79 min, tourniquet, spinal anesthesia, no TXA, cement used	Aspirin	5.7% (PE), 13.1% (DVT)	1.4	DVT

### Major bleeding events

Risk factors for MBE in the initial univariate analysis were preoperative use of chronic Warfarin (*p* < 0.001), older age (*p* < 0.001), higher BMI (*p* = 0.002), current or past smoking (*p* < 0.001), history of VTE (*p* < 0.001), hyper-coagulopathy (*p* < 0.001), higher ASA score (*p* < 0.001), Myocardial infarct (*p* < 0.001), heart failure (*p* = 0.002), atrial fibrillation (*p* < 0.001), complicated DM (*p* = 0.001), PUD (*p* < 0.001), CRF (*p* < 0.001), Sjogren syndrome (*p* = 0.015), chronic anemia (*p* = 0.009), varicose veins (*p* < 0.001), active and history of malignancy (*p*’s < 0.001), underlying fracture (*p* = 0.008), operative duration (*p* < 0.001), revision surgery (*p* < 0.001), cemented prosthesis (*p* < 0.001), tourniquet use (*p* < 0.001), direct lateral approach to the hip (*p* < 0.001), general anesthesia (*p* < 0.001), not using tranexamic acid (*p* < 0.001), allogenic blood transfusions (*p* < 0.001) and use of Warfarin for VTE prophylaxis (*p* < 0.001).

**Figure 6:**
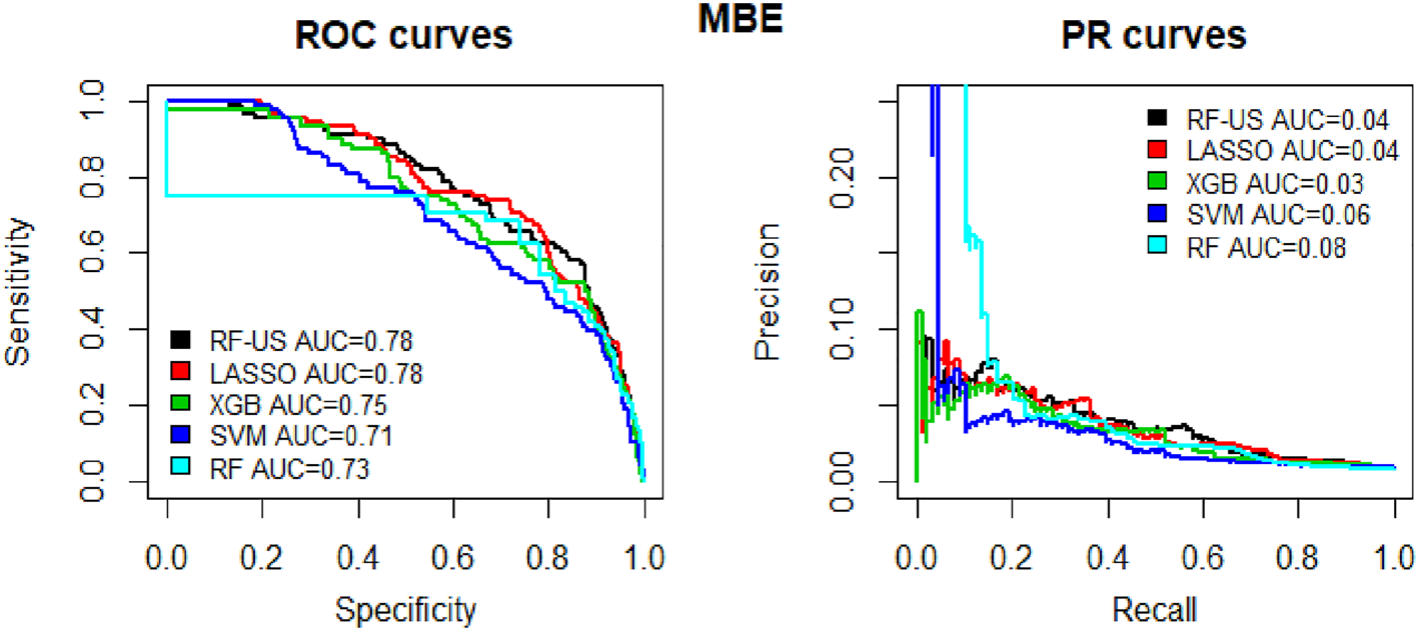
ROC curves for the various models that were assessed for MBE.

**Figure 7:**
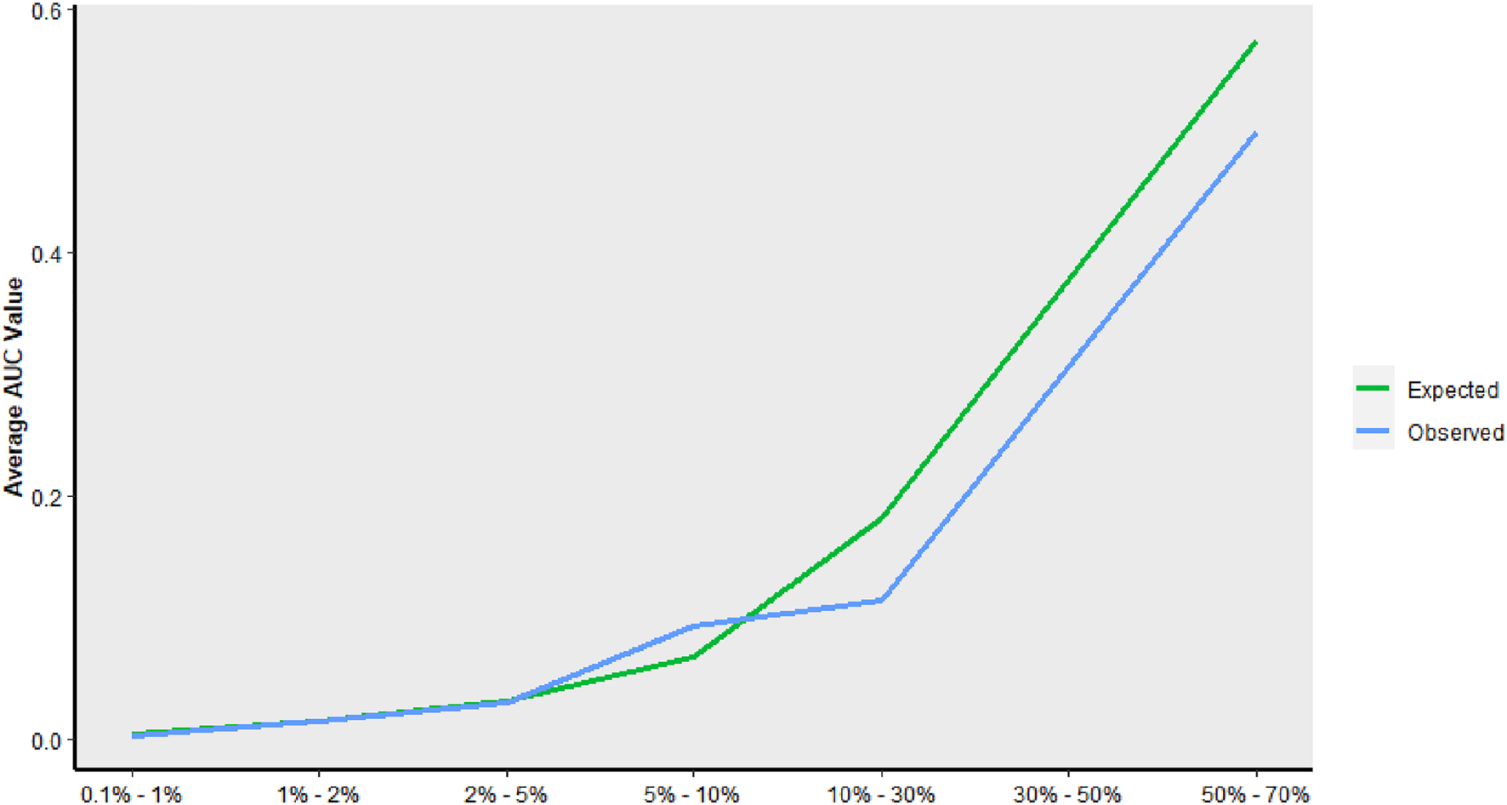
Predicted probability of major bleeding events and observed proportion stratified into groups based on risk.

The MBE models were tested using repeated cross-validation. Lasso analysis had the highest AUC (AUC-ROC 0.803 [SD 0.035]) on the testing set and was chosen for MBE algorithm development (Fig. 6 reflect performance on the validation cohort). Patients were grouped into categories according to predicted probability of MBE and the proportion of actual MBE in each category was examined; high agreement between expected and observed events was seen (Fig. 7).

**Figure 8:**
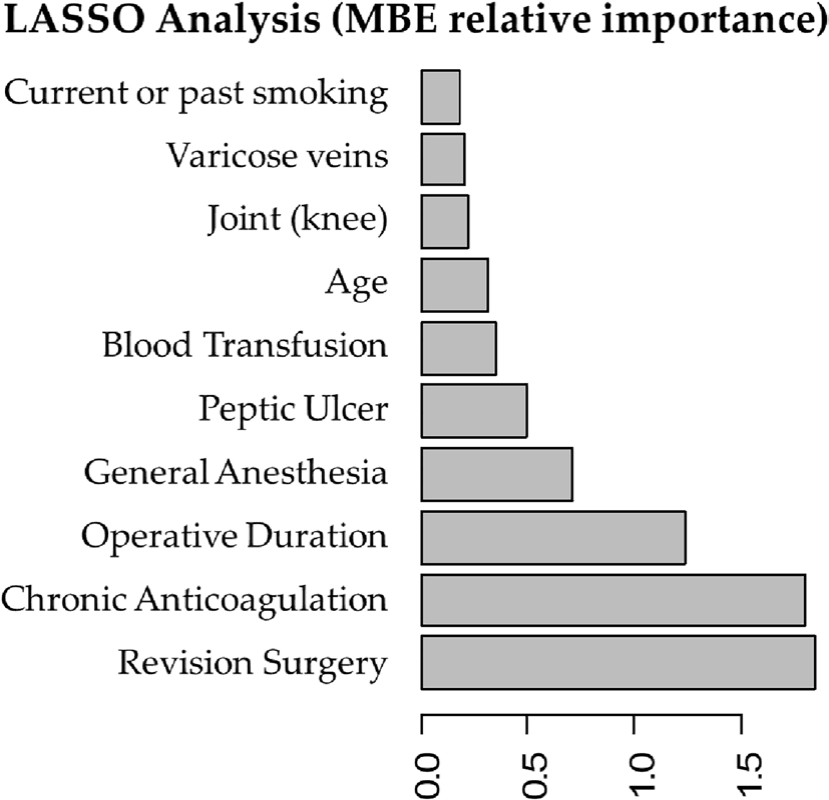
Top ten important predictors for major bleeding event (MBE) as captured by Lasso analysis.

Lasso analysis for the entire cohort showed that the 10 most important factors associated with MBE were the following (by order of importance): revision surgery, chronic use of warfarin preoperatively, operative duration, general anesthesia, peptic ulcer disease (PUD), allogenic blood transfusions, older age, knee joint, varicose vein and current or past smoking (Fig. 8). Examples of different clinical scenarios and the algorithm predictions are demonstrated in Table 2.

## Discussion

Due to the high morbidity and mortality associated with VTE and MBE, risk stratification prior to surgery is of great interest. Numerous studies have sought to identify specific risk factors that are associated with VTE and MBE^41,42^, and risk stratification models have also been proposed by prior studies reflecting its clinical importance^8,14–16^. The present study was designed and executed to take advantage of recent developments in machine learning algorithms that are patient-specific. We have successfully created an algorithm that not only accurately predicts VTE and MBE but also provides guidance on how different prophylactic measures may mitigate each individual’s risk. This study represents a major advancement in decision making prior to surgery and represents another major step towards patient-specific management.

Past studies have recognized multiple risk factors associated with VTE and MBE, including increasing age, previous VTE, revision surgery, cancer, type of procedure and more^43–45^. While the nature of the models used in this study were ‘black box’ which does not allow us to fully understand the decision trees generated to reach the final algorithm, looking at the random forest relative importance does provide insight into its decision making. All 10 of the most important variables that were pointed out for each one of the 3 outcomes—MBE, DVT and PE, have been previously reported as risk factors, providing reassurance that the algorithm is not only accurate, but consistent with accepted risk factors. One important finding was that while PE and DVT have many risk factors in common, one can gain from using different models in order to predict each of them and therefore we created and validated VTE and PE models separately.

In a recent systematic review of risk prediction scores for venous thromboembolism following joint replacement five observational cohort studies describing five risk scores were included^46^. The number of component variables in a single risk score ranged from 5 to 26. Three risk scores comprised 5–8 component variables. None of the studies reported calibration or discrimination statistics that can be directly compared to our model. Validation was lacking for all previous studies and they could be divided to ones in which scores are presented and evaluated within the same cohort or a holdout of patients^9,15^, and other studies specifically set out to validate an existing score or protocol^16,47–51^. Nam et al.^51^ were the only group prospectively evaluating their institutional protocol thus were able to take into account the influence of chemoprophylaxis in their evaluation. Using a simple protocol they categorized patients to “routine” (75.4%) and “high” (24.6%) risk groups and show 0.5% VTE rates in both. Whether their protocol does not capture high risk patients or the risk was mitigated by use of more aggressive anticoagulation in that group is debatable. In another study, Parvizi et al. used National Inpatient Sample (NIS) registry data to develop an individualized risk model for VTE which was based on 26 risk factors. The authors used scoring criteria to assess the performance of the model. The authors reported that their calibration curve showed a near perfect fit between the predicted VTE rate (using the risk model) and the actual rate of VTE in NIS data up to a 5% rate of VTE, beyond which point there was a clear divergence. Batelman et al.^16^ retrospectively evaluated the caprini and VTEstimator in a group of 363 TKA and THA patients. They failed to show an association between mean scores and risk for VTE. The study while interesting suffers from many methodological issues including small sample size and event rate (only 10 VTE), inability to assess the scores adequately due to missing data, and the evaluation of scores as continuous as opposed to categorical for risk stratification^52^. Krauss et al.^47^ compared a departmental protocol to the caprini score and showed that with a threshold of 10, the later was able to capture 7 out of 8 VTE compared to only 1 event that was captures using the departmental protocol. Notably, this threshold was evaluated and chosen to optimize the results of the caprini score within that specific cohort (using the youden index) and therefore does not reflect a true external validation of the score. More recently, Gold et al.^48^ in a cohort of 2155 TJA patients failed to find an association between high caprini scores both when evaluated continuously and categorically (with 11 as the threshold) when taking into account chemoprophylaxis in a multivariate analysis.

Several advantages make the present study stand out. The first is that the data that was used to develop the algorithm was derived from a single center and hence granular and contemporary. The Caprini score^47^ would automatically categorize patients undergoing TJA as high risk, however these surgeries have shifted tremendously over the years from having extended hospitalizations and limited postoperative weight bearing to becoming outpatient procedures with immediate mobilization. More and more patients receive spinal anesthesia, tranexamic acid, and aspirin as the primary chemoprophylaxis agent. These changes have reduced the rates of VTE during the last decade and our granular institutional data allowed us to capture these changes and take into account their influence^48^. A quick look at the univariate and relative importance analysis shows that modifiable factors such as tranexamic acid, type of anesthesia, and type of prophylaxis are important variables that previous studies/algorithms do not consider at all. The fact that these are modifiable variables is exciting as their influence could be tested in real time to examine whether they can change the individual patient’s risk. Previous works assumed that when patients have multiple risk factors and a calculated high risk they could benefit from more potent chemoprophylaxis—however recent studies have shown that is not the case which calls into question their practical use^49^. This algorithm could be used in everyday decision making prior to surgery and have an immense impact on treatment and hopefully lead to better outcomes and reduced VTE and MBE rates. Another important advantage is not only the assessment of VTE but also MBE that receives less attention in general but may also have a disastrous effect. Decisions that affect VTE result in a change in the MBE risk as well and vice versa, so the assessment should always include both outcomes.

This study is not without limitations. First, “black box” analyses as the ones used in this study are not easily interpretable and it remains unclear how the model predicts outcome. While relative importance and cross validation shed some light on what the model relies on more heavily, we are still left with some uncertainty. Second, while analyzing a cohort of almost 40,000 from a single institution, we may still have been underpowered to detect influence of some of the variables due to the low event rates of both VTE and MBE. This may have reduced the performance of all three models. Third, we were unable to detect whether the DVT cases were proximal or distal. One could assume the majority were proximal as all received treatment, however we cannot say for sure. Our conclusion therefore apply to clinically overt cases. Fourth, the design was retrospective hence mistakes in data collection and extraction may have occurred. Fifth, the model includes variables that are not always known prior to surgery and can only be assumed (such as operative time), limiting its utility for prospective risk stratification. Finally, there are many possible variables that were not assessed in the present study and their inclusion may have improved the predictive capabilities. Future studies to incorporate these factors into the algorithm may further refine this tool.

To conclude, we successfully created and validated an easy-to-use, practical and accurate tool for predicting VTE and MBE following TJA (which can be accessed through the following link: https://icmphilly.wordpress.com/ortho-applications). This algorithm can and should be used by clinicians in practice to improve decision making and patient counseling. There is currently an international multicenter study on the way to validate our findings externally.

## Supplementary Information

Supplementary Tables.

## Data Availability

The datasets generated during and/or analysed during the current study are not publicly available. Please contact research@rothmanortho.com to request data.
