# Supplementary material for: Using machine learning to predict venous thromboembolism and major bleeding events following total joint arthroplasty
Source: Sci Rep. 2023 Feb 7;13:2197. doi: 10.1038/s41598-022-26032-1 (PMC9905066; doi:10.1038/s41598-022-26032-1)
Supplement: Supplementary file 1 — Supplementary Tables. [file 41598_2022_26032_MOESM1_ESM.docx]

**Supplementary Table 1.** Keywords included and excluded in the search criteria to capture patients with history of a coagulopathy.

| **INCLUDED TERMS** | **EXCLUDED TERMS** |
| --- | --- |
| HEMOPHILIA | DISEASE |
| VON WILLEBRAND DISEASE | BLEED |
| SICKLE CELL ANEMIA | PT RX |
| THROMBOCYTOPENIA | PT REPORTS |
| DISSEMINATED INTRAVASCULAR COAGULATION (DIC) | PT PULSES ARE PALPABLE |
| COAGULOPATHY | PT EVAL |
| INR | PT SCRIPT |
| PTT |  |
| EXCESSIVE BLEEDING |  |

**Supplementary Table 2.** Keywords included and excluded in the search criteria to capture patients with history of a hypercoagulable state

| **INCLUDED TERMS** | **EXCLUDED TERMS** |
| --- | --- |
| FACTOR V LEIDEN | CYST |
| HYPERCOAG | PT RX |
| HOMOCYST | PT REPORTS |
| FACTOR 5 | PT PULSES ARE PALPABLE |
| FACTOR V | PT EVAL |
| LEIDEN | PT SCRIPT |
| INR | PT |
| PTT |  |
| EXCESSIVE BLOOD CLOTTING |  |

**Supplementary Table 3.** Keywords included and excluded in the search criteria to capture patients with DVT or PE.

| **INCLUDED TERMS** | **EXCLUDED TERMS** |
| --- | --- |
| BLOOD CLOT | ASPIRATION |
| CLOT | LET US KNOW |
| LEG SWELLING | FRACTURE |
| SWOLLEN LEG | MI |
| BLOOD THINNER | MYOCARDIAL INFARCT |
| CALF | INFARCT |
| PRESSURE | HEART ATTACK |
| ULTRASOUND | FALL |
| US | PAIN |
| DOPPLER | CONSTIPATION |
| VENOGRAM | NAUSEA |
| SONOGRAM | CHF |
| EMERGENCY DEPARTMENT | HEART FAILURE |
| ED | MI |
| EMERGENCY ROOM |  |
| ER |  |
| BLOOD CLOT |  |
| CLOT |  |
| LUNG |  |
| PULMONARY EMBOLISM |  |
| PE |  |
| SPIRAL CT |  |
| CT |  |
| VQ SCAN |  |
| WELLS SCORE |  |
| DIMER |  |
| CHEST X-RAY |  |
| CHEST X RAY |  |
| CHEST RADIOGRAPH |  |
| PULMONARY ANGIOGRAPHY |  |
| RAPID BREATHING |  |
| MEMOPTYSIS |  |
| SOB |  |
| SHORTNESS OF BREATH |  |
| EMERGENCY DEPARTMENT |  |
| ED |  |
| EMERGENCY ROOM |  |
| ER |  |
| CHEST PAIN |  |
| BLOOD THINNER |  |
| HEPARIN |  |
| LOW MOLECULAR WEIGHT HEPARIN |  |
| LMWH |  |
| ENOXAPARIN |  |
| COUMADIN |  |
| WARFARIN |  |
| RIVAROXABAN |  |
| XARELTO |  |
| APIXABAN |  |
| ELIQUIS |  |
| DABIGATRAN |  |
| PRADAXA |  |
| EDOXABAN |  |
| SAVAYSA |  |

**Supplementary Table 4.** Keywords included and excluded in the search criteria to capture patients with a major bleeding event.

| **INCLUDED TERMS** | **EXCLUDED TERMS** |
| --- | --- |
| GI BLEED | GASTROINTESTINAL INFECTION |
| GASTROINTESTINAL BLEED | GASTROINTESTINAL CONSTIPATION |
| INTESTINAL BLEED | CONSTIPATION |
| INTESTINAL HEMORRHAGE | GI INFECTION |
| GI HEMORRHAGE | GI CONSTIPATION |
| GASTROINTESTINAL HEMORRHAGE | WOUND BLEEDING |
| RECTAL BLEED | BLEEDING FROM WOUND |
| INTERNAL BLEED | INTRAOPERATIVE BLEED |
| MELENA | INTESTINAL |
| VOMITING BLOOD | INCLUDING BLEEDING |
| BLOOD VOMIT | NOBLEEDING PROBLEMS: NO |
| BLOOD IN THE STOOL | NO BLEEDING |
| BLOOD IN STOOL |  |
| BLOODY STOOL |  |
| GASTROINTESTINAL |  |
| #GI# |  |
| BLEEDING |  |
| HEMORRHAGE |  |
| #BRAIN# |  |
| HEMORRHAGIC SHOCK |  |
| RUPTURED BLOOD VESSEL |  |
| CEREBRAL HERMORRHAGE |  |
| BLOOD IN URINE |  |
| BLOODY URINE |  |
| HEMATURIA |  |
| BLOODY IN URINE |  |
| BLOODY IN THE URINE |  |
| MAJOR BLEEDING |  |
